# Sphingolipids and Mitochondrial Dynamic

**DOI:** 10.3390/cells9030581

**Published:** 2020-03-01

**Authors:** Lais Brigliadori Fugio, Fernanda B. Coeli-Lacchini, Andréia Machado Leopoldino

**Affiliations:** Department of Clinical Analyses Toxicology and Sciences, School of Pharmaceutical Sciences of Ribeirão Preto, University of São Paulo, Av. do Café s/n, 14040-903 Ribeirão Preto, SP, Brazil; lais.fugio@usp.br (L.B.F.); fbcoeli@gmail.com (F.B.C.-L.)

**Keywords:** ceramide, sphingosine, mitophagy, fission, fusion, mitochondria

## Abstract

For decades, sphingolipids have been related to several biological functions such as immune system regulation, cell survival, and proliferation. Recently, it has been reported that sphingolipids could be biomarkers in cancer and in other human disorders such as metabolic diseases. This is evidenced by the biological complexity of the sphingolipids associated with cell type-specific signaling and diverse sphingolipids molecules. As mitochondria dynamics have serious implications in homeostasis, in the present review, we focused on the relationship between sphingolipids, mainly ceramides and sphingosine-1-phosphate, and mitochondrial dynamics directed by fission, fusion, and mitophagy. There is evidence that the balances of ceramides (C18 and C16) and S1P, as well as the location of specific ceramide synthases in mitochondria, have roles in mitophagy and fission with an impact on cell fate and metabolism. However, signaling pathways controlling the sphingolipids metabolism and their location in mitochondria need to be better understood in order to propose new interventions and therapeutic strategies.

## 1. Introduction

Sphingolipids are a class of complex lipids with several functions, including membrane structure and intracellular and extracellular signaling, associated with a large number of processes. Their deregulations have been broadly described in human diseases with a strong potential for therapeutic strategies. Fingolimod (FTY720), the first oral treatment for patients with multiple sclerosis, approved by Food and Drug Administration (FDA) in 2010, is an analog of sphingosine with an immunosuppressant action [1]. Fingolimod application still has been investigated in clinical trials for other medicine proposals (<https://clinicaltrials.gov/ct2/home>) such as stroke, cerebral edema, schizophrenia, neurodegeneration, renal patients, cancer (breast carcinoma and glioblastoma), and multiple sclerosis patients. These points highlight the relevance of the balance of sphingolipids in normal cell functions as egress of lymphocytes from lymph nodes [1].

**Figure 1:**
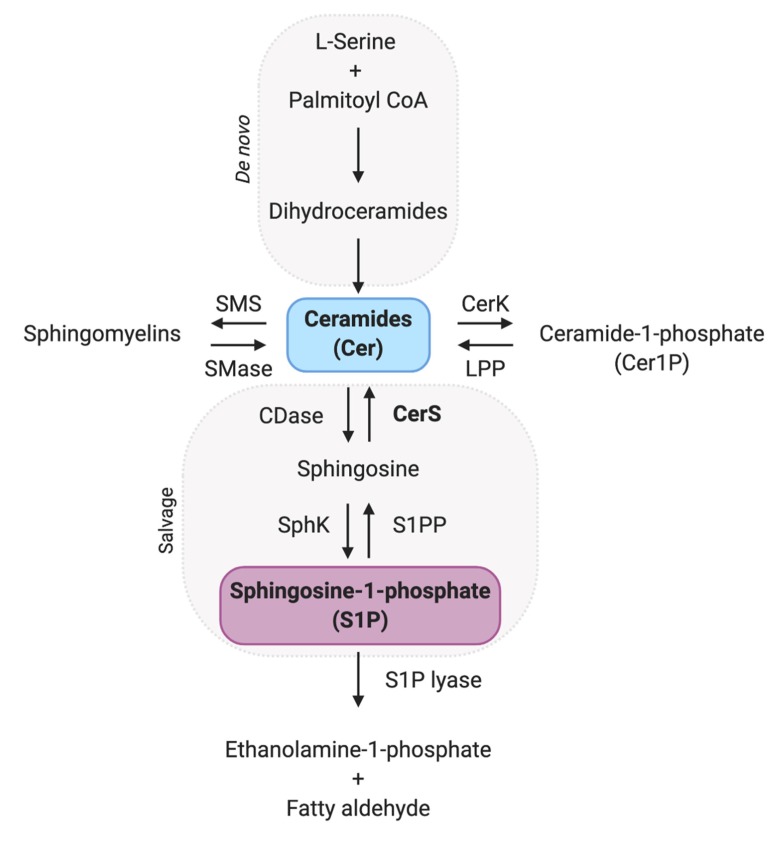
Sphingolipids’ metabolism pathways, including the enzymes and molecules related to ceramides and S1P levels. CoA: coenzyme A; SMS: sphingomyelin synthase, SMase: sphingomyelinase; CerK: ceramide kinase; LPP: lipid phosphate phosphatase; CDase: ceramidase; CerS: ceramide synthase; SphK: sphingosine kinase; S1PP: sphingosine-1-phosphate phosphatase; S1Plyase: sphingosine-1-phosphate lyase.

Cellular levels of sphingolipids (SL) can be altered either by the delivery via lipoproteins (high-density lipoprotein, low-density lipoprotein and very low-density lipoprotein) or by serum albumin. Besides, red blood cells and platelets can release sphingolipids, modifying their local levels as well as extracellular and intracellular enzymes [2,3]. In general, the diet affects lipids and sphingolipid profiles, as observed in high-fat diet, obesity, and type 2 diabetes [4,5]. Serine, a substrate to ceramide synthesis, when absent, caused mitochondria fragmentation reversed by supplementation with C16:0-ceramide [6] and intermittent fasting associated with high intensity-intermittent exercise up-regulated F_o_F_1_ adenosine triphosphate (ATP) synthase activity in apparent association with an increase in mitochondria mass [7,8]. These results appoint the relation between specific sphingolipids with mitochondria though the molecular mechanism and signaling is not fully understood. In Figure 1, we highlighted sphingolipids and enzymes involved in their metabolism [9].

The effects of sphingolipids in mitochondria are highly relevant because the organelle is responsible for bioenergetics, and alterations in mitochondrial functions have been associated with cell death signaling, autophagy, chemoresistance, and cellular stress [10,11]. Besides, sphingolipids can modify mitochondrial morphology by biophysical effects on mitochondrial outer (OMM) and inner membranes [12]. Mitochondria sphingolipids’ composition determined by mass spectrometry identified 31 sphingolipids as ceramides, glyceroceramides (GlcCer), sphingomyelins (SM), and gangliosides (Gan) [7]. Mitochondria also contain other lipids such as glycerophospholipids (such as phosphocholine (PC), phosphoethanolamine (PE), phosphoglycerol (PG), and cardiolipin (CL)), fatty acyl (FA), and glycerolipids (GL, including triacylglycerol (TG)). Outer (OMM) and inner mitochondrial membranes are constituted mainly by phospholipids with asymmetric distribution, suggesting their importance to mitochondria function [13]. However, ceramides accumulate in OMM and form channels affecting mitochondria function and homeostasis, and together with cardiolipin, can regulate mitophagy [14,15,16]. The metabolism and change of sphingolipids in organelles, membranes, or cellular compartments impact cell biology, and consequently can contribute to pathological conditions as obesity, diabetes, cancer, stroke, oxidative stress, aging, and neurodegeneration [17,18,19].

Two processes, biogenesis and mitophagy, rigorously control the mitochondrial mass and homeostasis. In the present review, we focused on the relationship between sphingolipids, mainly ceramides and sphingosine-1-phosphate, and mitochondrial dynamics directed by fission, fusion, and mitophagy.

## 2. Ceramides and Mitochondrial Dynamics

Ceramides are molecules composed of a sphingoid base with 18 carbons linked by an amide bond to a fatty-acyl chain [20]. Ceramides’ production involves at least three pathways upon activation in different cellular compartments and is an intermediate to other complex sphingolipids [21,22]. De novo pathway depends on fatty-acyl CoA and ceramide synthases (CerS1-6), which are integral endoplasmic reticulum (ER) membrane proteins, each of them synthesizes ceramides with acyl chain lengths from 14 to 26 carbons [23] with distinct biological roles [22]. For example, CerS1 generates C18-ceramide, mediating cell death and tumor suppression in acute myeloid leukemia (AML) and head and neck squamous cell carcinoma (HNSCC) [24,25], while CerS6 has an antiapoptotic role in ER stress responses and produces mainly C16-ceramide [26].

Ceramide is a bioactive sphingolipid, well-known for regulating mainly apoptosis, autophagy, and senescence [27,28,29]. There is evidence suggesting that ceramide acts locally in mitochondria rather than in other organelles. Constructs targeting bacterial sphingomyelinase (SMase) protein were transfected in individual cellular compartments of MCF-7 breast cancer cells, increasing SMase activity and, accordingly, total cellular ceramide levels. Interestingly, only mitochondrial SMase-derived ceramide activated apoptosis in these cells, whereas overexpression of SMase-derived ceramide in ER, Golgi, and plasma membrane did not activate [30]. However, it was already reported that exogenous ceramide activates the ER stress in human salivary adenoid cystic carcinoma activating pro-apoptotic signaling pathways [31]. Moreover, there are mitochondrial-associated ER membranes (MAMs) involved in protein, Ca^2+^ homeostasis, and lipid transport between ER and mitochondria, influencing the distribution and local concentration of the sphingolipids that affect mitochondrial function to cell survival or death [32]. Deficiency in acid sphingomyelinase activity, which causes accumulation of sphingomyelin and significantly reduces the levels of ceramide, has been reported to prevent ionizing radiation-induced apoptosis in lymphoblasts from Nieman–Pick disease patients and in lung tissues from acid ceramidase knockout mice [33], indicating that, independently of the metabolic pathway to produce ceramides, their accumulation seems to promote apoptosis.

Recently, several ceramide species have been reported as elevated in breast cancer tissues when compared with control tissues and associated with less aggressiveness [34]. Ceramide-mediated cell death has been related to mitochondrial dysfunction in many cell types, such as cardiomyocytes [35], glioblastoma [36], and lung epithelial cells [37]. We can speculate that a high level of ceramide may impair cancer cell proliferation and survival [38,39], consequently promoting a favorable impact on patient survival. However, new studies will be necessary to expand our knowledge on the profile of ceramides and other sphingolipids in cancer patients and to explore their application as biomarkers for prognosis and potentially for therapy response in several types of cancer and other pathologies. A limitation of the sphingolipids studies has been related mainly to the analytical method and costs to perform the identification and quantification when a home lipidomics facility is not available. This seems to be a barrier to the future application of lipidomics in the clinical routine that we need to break down, proving the feasibility of analyzing those molecules and the implications of sphingolipids in health.

### Ceramides and Mitophagy

Autophagy is a catabolic process conserved in the cells that degrades cytoplasmic components in the lysosome [40]. This components ranges from proteins to organelles [40]. For example, damaged mitochondria are targeted to autophagosomes, which are trafficked to lysosomes for degradation in a process termed mitophagy [41].

Mitophagy, a tightly regulated process for mitochondria degradation, can activate a type of programmed cell death independent of apoptosis, also named lethal mitophagy, dependent on C18-ceramide generated by CerS1 [42]. It has been reported in head and neck squamous cell carcinoma that CerS1/C18-ceramide binds to LC3B-II protein, which binds ceramide to the outer mitochondrial membrane (OMM). The Ile35 and Phe52 residues of the hydrophobic domain of LC3B, as well as PE lipidation, are required for the interaction with ceramide in OMM, suggesting a novel role for ceramide as an anchoring molecule for LC3B-II autophagolysosomes, favoring lethal mitophagy [42,43]. The regulation of ceramide-induced mitophagy is downstream to mitochondrial fission, mediated by dynamin-related protein (Drp1), which participates in fission and promotes membrane scission of mitochondria [44]. Drp1 knockdown affects ceramide distribution in OMM, preventing mitophagy. Besides, CerS1/C18-ceramide can induce tumor suppression in model of head and neck squamous cell carcinoma (UM-SCC-22A) xenograft tumor formation [42]. The C18-ceramide could be defined as an essential molecule to trigger mitophagy; alterations in enzymes of the metabolism as well as nutrition can modulate its production inside the cells, affecting mitochondria and cell functions.

Besides, CerS6/C16-ceramide generation did not induce mitophagy, but treatment with C16-pyridinium ceramide induced mitophagy owing to its accumulation in the mitochondria. These results suggested that not only the acyl chain length is essential to determine the biological effect of ceramide species in mitophagy, but also their subcellular localization [42].

**Figure 2:**
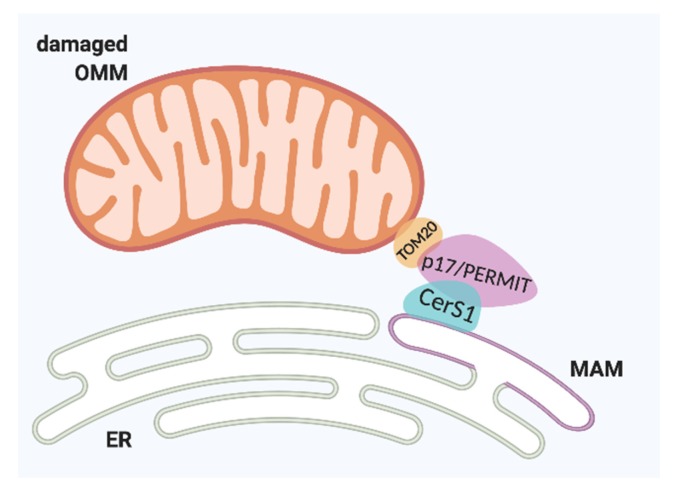
Trafficking of CerS1 by endoplasmic reticulum (ER) mitochondria-associated membranes (MAMs). CerS1: *ceramide synthase 1, p17/PERMIT:* protein that mediates ER-mitochondria trafficking; TOM20: translocase of outer mitochondrial membrane 20. OMM, outer mitochondrial membrane.

Recently, the mechanism underlying the translocation of CerS from ER to mitochondria in UM-SCC-22A has confirmed the cellular distribution as a critical event. A recently identified protein, p17/PERMIT, mediates ER-mitochondria trafficking of newly translated CerS1 from the ER surface to OMM through mitochondria-associated membranes (MAMs) to induce mitophagy, as illustrated in Figure 2 [45]. The mitochondrial translocation of both CerS1 and p17/PERMIT occurs via the interaction between the residues 28RYE30 and R68, respectively [45]. The signaling initiated by stress conditions activates Drp1 and induces mitochondrial fission, accompanied by mitochondrial membrane damage. It is followed by p17/PERMIT-CerS1 complex translocation to the damaged OMM, which showed that PERMIT residues 1 to 25 are necessary for interaction with TOM20. Consequently, CerS1/C18-ceramide generation induces LC3B-II targeting autophagolysosomes to mitochondria, leading to mitophagy-mediated cell death [42,45].

Another example has been reported in human papillomavirus (HPV +) HNSCC cells, where HPV early protein 7 (E7) induces ceramide-mediated lethal mitophagy in response to cisplatin, by targeting retinoblastoma protein (Rb) [46,47]. Besides that, cisplatin increased the accumulation of CerS1 in mitochondria, suggesting that HPV-E7 enhances lethal mitophagy via CerS1/C18-ceramide. Xenograft tumors in severe combined immunodeficiency (SCID) mice in response to chemotherapy increased CerS1 and CerS6 mRNA expression in HPV(+), but not in HPV(−). Drp1 knockdown prevented cisplatin-induced mitophagy by HPV-E7, confirming the mechanism. Moreover, the cell death in HNSCC HPV-E7(+) cells is mediated by cytoplasmic E2F5 activation by targeting RB. Finally, the mechanism proposed is that E2F5 binds to Drp1 and induces its translocation to mitochondria (mitochondrial fission), which, in turn, leads to ceramide-dependent mitophagy [47].

However, CerS6 involvement in autophagy and cisplatin-induced mitochondrial fission has been demonstrated in OSCC cells. CerS6 mRNA is reduced in cisplatin-resistant OSCC cells and its overexpression in these cells increased their susceptibility to cisplatin, causing mitochondrial fission, decreased LC3 conversion and p62 degradation, and apoptosis accompanied by decreased mitofusin 2 (MFN2) and increased calpain activity [48]. Previously, the degradation of mitofusin 2 (MFN2) by calpain-inducing mitochondrial dysfunction in motor neurons has been shown, suggesting this as a pathway [49]. As a proof-of-concept, the sensitivity to cisplatin using an OSCC xenograft tumor formation assay has been reversed by the reduction of CerS6 [48]. Together, this evidence indicates that CerS6 is involved in the response to cisplatin resistance in OSCC cells and confirms that mitochondrial dynamics is an essential mechanism in cell fate potentially associated with mitophagy, autophagy.

In acute myeloid leukemia (AML), it has been shown that FLT3-ITD (mutant Fms-like tyrosine kinase 3–internal tandem duplication) downregulates the CerS1/C18-ceramide axis, inducing resistance, whereas its molecular or pharmacological targeting reactivates CerS1/C18-ceramide generation, accompanied by mitophagy and cell death both in AML cell lines and blasts obtained from FLT3-ITD1 patients and in xenograft models in vivo [24]. Furthermore, LCL-461, a soluble C18-ceramide analog drug, accumulates in mitochondria and induces lethal mitophagy in AML cells expressing FLT3 mutations and in blasts obtained from FLT3-ITD1 AML patients [24]. Interestingly, it has been reported that the dephosphorylation-dependent activation of Drp1 at S637 by protein kinase A (PKA) inhibition is a signal to translocate CerS1 to mitochondria [24], where C18-ceramide recruits autophagosome to lethal mitophagy [42]. These data suggest the accumulation of C18-ceramide in mitochondria of cancer cells as a indicative factor of good prognosis in cancer patients.

Looking for another model, we can highlight previous works that reported an elevation in ceramides levels in the myocardium associated with cardiac dysfunction in various models of cardiomyopathy, proposing an essential role for ceramides in the pathologies [50,51,52]. CerS2-specific species are increased in the myocardium of high fat diet-fed mice, and CerS2 overexpression, but not CerS5, induced mitophagy, while CerS2 knockdown prevented palmitate-induced mitophagy [35]. Opposite to C18-ceramide, CerS2-derived ceramides (very-long-chain ceramides) caused mitochondrial dysfunction, leading to a protective mitophagy. This suggests that the impact caused on mitochondrial function, mainly mitophagy, is dependent on cell type and ceramide chain-length. Further studies are necessary to understand how the ceramide synthases are regulated and which ceramide species are controlling mitophagy in different cell types and conditions. It is fundamental to propose new inhibitors for ceramide synthases and other enzymes as well as the therapeutic use of sphingolipids analogs. For example, Fingolimod is proposed to stimulate autophagy, necrosis, necroptosis, and apoptosis with a protective and lethal impact in some cell types [53,54,55], and it has a massive impact on immune system response [56,57,58]. How much is this effect ceramide species-dependent via mitochondria?

The balance between mitochondrial fusion and fission has several implications in health and pathological conditions. In general, fusion generates healthy mitochondria, whereas fission removes non-functional organelles [59]. Interestingly, it had already been suggested that ceramides regulate mitochondrial fusion proteins and induce fission in different disease models [48,60,61,62]. C2-ceramide treatment (a permeable ceramide analog) stimulates mitochondrial fission by increasing Drp1 and Fis-1 protein levels in mitochondria and leads to apoptosis in neonatal rat cardiomyocytes [61]. Accordingly, it has been shown that C2-ceramide induces reactive oxygen species (ROS) and increases Drp1 expression in mouse myoblast cell line C2C12 [62].

Besides, it has been described that increased cell death and autophagy in preeclampsia (PE) is in part ceramides-dependent, once C16-ceramide and C18-ceramide levels are increased in PE placentae compared with age-matched control placentae [63]. This elevation of ceramide in mitochondria has been associated with BOK (pro-apoptotic member of Bcl-2 family) recruitment to OMM accompanied by p-DRP1-dependent mitochondrial fission, increasing mitophagy [60]. Summarizing, these mitochondrial dynamic events accompanied by a high level of ceramides and enhancing fission contribute to excessive autophagy and cell death, which is commonly reported in PE.

Recently, it has been elucidated that mice with CerS6 deficiency have protection against high fat diet (HDF)-induced liver dysfunction and mitochondrial fragmentation, which improved insulin sensitivity [64]. Moreover, CerS6-derived C16:0 sphingolipids, but not CerS5-derived, can interact with the mitochondrial fission factor (Mff) and promote mitochondrial fission and insulin resistance in obesity [64]. This difference between sphingolipids may be because of the ability to regulate CerS protein levels and transportation, and consequently the subcellular pools of ceramide species. However, the molecular interaction between C16:0 ceramide and Mff remains to be elucidated.

**Figure 3:**
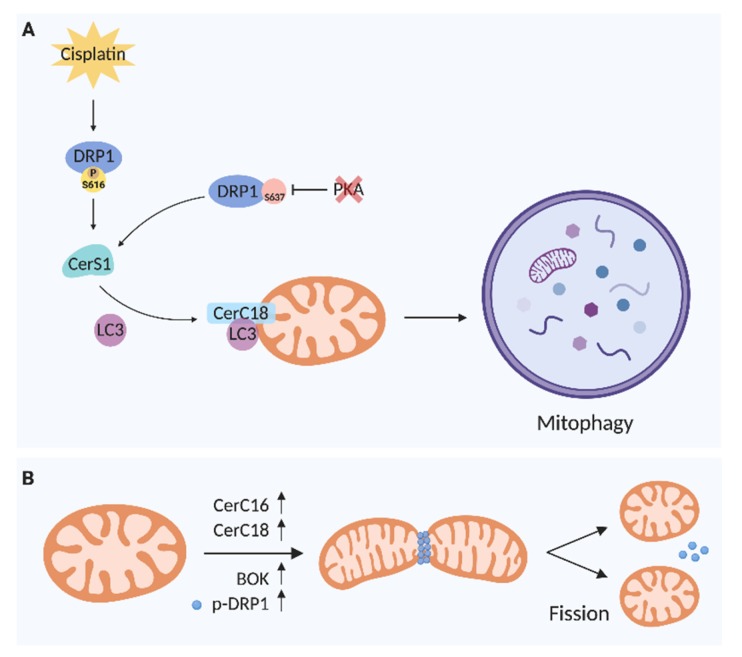
Ceramides involved in mitochondrial dynamics. **A**. Mitophagy. **B**. Mitochondrial fission. CerS1: ceramide synthase 1; DRP1: dynamin-related protein 1; p: phosphorylated; PKA: protein kinase A; BOK: Bcl-2 related ovarian killer; LC3: microtubule-associated protein1 light chain 3.

In Figure 3, we summarized the evidence supporting the role of ceramides in mitochondria dynamics. Therefore, the way by which the metabolism and balance of sphingolipids are regulated to control fission, fusion, and mitophagy still needs to be better defined.

## 3. Sphingosine-1-phosphate (S1P) and Mitochondrial Dynamics

Today, sphingosine-1-phosphate (S1P), described by the Spiegel group in 1991 [65], is a well-known and broadly studied bioactive sphingolipid. Its signaling has been associated with diverse biological functions and cell-type specificities such as proinflammatory [66] and proliferative effects [38]. S1P is produced from intracellular ceramides by ceramidases and degraded by S1P phosphatases and S1P lyases [22]. The enzyme ceramidase converts the ceramide into sphingosine, which is subsequently phosphorylated by sphingosine kinases (SphK) to produce S1P. There are two SphKs isoenzymes, SphK1 and SphK2 [67,68]. The SphK2 has been identified in the endoplasmic reticulum, nuclei and mitochondria. Activated SphK1 is directed to the plasma membrane (PM), where it elevates the level of S1P, favoring its transport to the outside of the cells via spinster homolog 2 (SPNS2) [69], major facilitator superfamily transporter 2b (Mfsd2b) [70], and ATP-binding cassette (ABC) transporters such as ABC1 [71]. S1P signaling is transduced via specific heterotrimeric G-protein-coupled receptors named S1PR 1–5 [72]. The expression of S1PRs is cell-type specific, contributing to diverse functions reported by S1P and variability of cell responses. This biological complexity should be explored because it offers opportunities to develop specific therapeutic strategies.

As mentioned, the function of S1P is variable according to the cell type and to the location where it is accumulated. For example, S1P present in the nucleus is produced by SphK2 activity and acts as a histone deacetylase inhibitor, controlling specific genes involved in the metabolism of sterol and lipids in hepatocytes upon conjugated bile acids and S1P receptor 2 (S1PR2) signaling [73]. Besides, an animal model used to study the impact of S1P accumulation in the brain (mainly hippocampus) showed its relation with memory function [74]. These works are suggesting a potential application for sphingosine analogs as a therapeutic intervention for liver and brain disorders. However, the full mechanism regulating the S1P and its potential impact in mitochondria dynamics in both the liver and brain have not been reported and other models have not been studied.

There is evidence reporting that S1P signaling regulates mitophagy during terminal erythroid differentiation. S1P promotes the regulation of Pink1-p62- and Nix/Bnip3l-mediated mitophagy and the role of Nix/Bnip3l in the regulation of mitophagy in erythropoiesis has been validated in a knock-out mice model [75]. When SphK1 was inhibited, a reduction in Pink1 expression was observed, and consequently suppressed p62 recruitment to mitochondria in late erythroblasts. These cells with elevated ROS levels presented a reduction in mitochondria clearance, resulting in increased apoptotic erythroid cells. This mechanism proposes that S1P contributes to terminal differentiation through the regulation of Pink1-p62- and Nix/Bnip31-mediated mitophagy [76]. The role of S1P in mitochondrial homeostasis indicates that the unbalance of sphingolipids and/or their signaling as transductors can be a disturbance of health.

### Sphingosine-1-phosphate (S1P) and Mitochondrial Fission and Fusion

Fission has been reported as a mechanism that precedes both apoptosis and mitosis. One example is the essential role of fission during asymmetric division and maintenance of the stem cell properties [77]. Interestingly, during stem cell differentiation, the mito-fission directs the newly formed mitochondria to self-renewed stem cells, while the old mitochondria will be kept in differentiated cells. Mitophagy is an event necessary for cleaning damaged mitochondria and recycling molecules, but in certain conditions when the damage is above the threshold, cell death will be activated. The opposite process controlling mitochondria morphology and structure and cell fate is mito-fusion, a known mechanism to exchange molecules between mitochondria and mitochondrion-nucleus, commonly active in differentiated cells. This process requires the coordination of multiple interacting factors such as mitofusins Mfn1/Mfn2 [78] and optic atrophy 1 (OPA1) [79].

Drp1 protein, a central regulator of fission, bears four domains: N-terminal guanine nucleotide-binding proteins (GTP)-binding, middle, insert B, and C-terminal GTPase effector (GED) [80]. Insert B acts in the regulative process of fission by binding the target mitochondrial membrane [80]. In cardiomyocyte, it has been shown that Ras homolog family member A (RhoA) activation regulates Drp1. The endogenous activation of RhoA by G protein-coupled recpetor under S1P stimuli or with S1PR3 agonists increases Drp1 phosphorylation and its mitochondrial translocation in a RhoA and ROCK-dependent manner [81]. Interestingly, RhoA activation does not lead to mitochondrial membrane depolarization or opening of the mitochondrial permeability transition pore. Albeit, S1P stimulation protected cardiomyocytes under oxidative stress against the loss of mitochondrial membrane potential [81]. This evidence supports an essential role for S1P, as a bioactive sphingolipid, in mitochondrial homeostasis.

In human renal glomerular endothelial cells (HRGECs), when S1PR2 was blocked by its antagonist (JTE-013), the reduction of RhoA, ROCK1, and Drp1 expressions was observed, while inhibiting ROCK1 led to Drp1 downregulation [82]. Surprisingly, an S1PR2 antagonist was able to reverse the high glucose-induced dysfunction in HRGECs. Moreover, ROCK1 was identified as a critical factor connecting S1PR2 to fission and dysfunction through Drp1. Therefore, S1PR2 activates the RhoA/ROCK1/Drp1 signaling pathway in HRGECs [82]. These data provide the first evidence that S1PR2 exerts multiple effects on the fission and dysfunction of mitochondria induced by high glucose in HRGECs, which in turn contribute to endothelial dysfunction [82].

Interestingly, in kidney tubule cells, S1PR1 seems to be a suitable target for maintaining mitochondrial integrity [83]. As the treatment with cisplatin causes nephrotoxicity in one-third of patients and often limits its usage [84,85], an alternative could be an adjuvant therapy to protect the cytotoxicity effects in non-target tissues. Recently, it has been shown that a sphingosine analog, Fingolimod, can attenuate acute kidney injury induced by cisplatin. Fingolimod showed efficiency in attenuate kidney ischemia-reperfusion injury by directly activating S1PR1 on proximal tubule cells, while S1PR1 overexpression induces an imbalance between fission and fusion [83]. Also, the expression of mitochondrial genes (Mitofusin 1; Mitofusin 2; Optic atrophy 1) was increased in S1PR1 overexpressing cultured mouse proximal tubule cells (TKPTS) cells, while S1P1-deficient primary tubule cells showed a reduction of the genes [83]. These results open a new perspective to explore sphingosine analogs as potential adjuvant medicine in cancer treatment based on S1P/S1PR signaling and mitochondria function.

S1P has been related to the regulation of glucose-stimulated insulin secretion in mouse insulinoma 6 (MIN6) cells and pancreatic islets [86]. Also, the loss of SphK was associated with β-cell death in obese mice [87], being the relation between S1P and prohibitin (PHB) linked to the maintenance of β-cell function and survival [88]. The knockdown of SphK2 and S1P reduction decreased glucose-stimulated insulin secretion and reduced the expression of PHB, a regulator of mitochondrial metabolism [88]. Accordingly, when the MIN6 cell line was treated with a sphingosine kinase inhibitor, the expression of OPA1 and Mfn1 also decreased. These data suggest that S1P plays an essential role in maintaining mitochondrial homeostasis. Besides that, it has been shown that knockdown of SphK1 does not affect PHB expression, while cellular S1P levels were drastically decreased by SphK2 knockdown, suggesting that SphK2 is a major regulator of cellular S1P levels in β-cells [88].

It was also reported that S1P, produced in mitochondria by SphK2, binds with specificity and high affinity to PHB2, a conserved protein implicated in regulating mitochondrial function and localized to the inner mitochondrial membrane (IMM) [89]. This association is involved in the assembly of the electron transport chain and mitochondrial respiration, identifying another function to SphK2 and S1P in mitochondrial dynamics [89]. Moreover, another group revealed that S1P lyase overexpression increases the expression of PHB2, preventing cell death by the maintenance of Ca^2+^ homeostasis and cytokine-induced mitochondrial and ER stress in insulin-secreting cells [90]. These findings show that both enzymes, SphK2 and S1P lyase, have an important role in mitochondria homeostasis and regulate mitochondrial dynamics.

**Figure 4:**
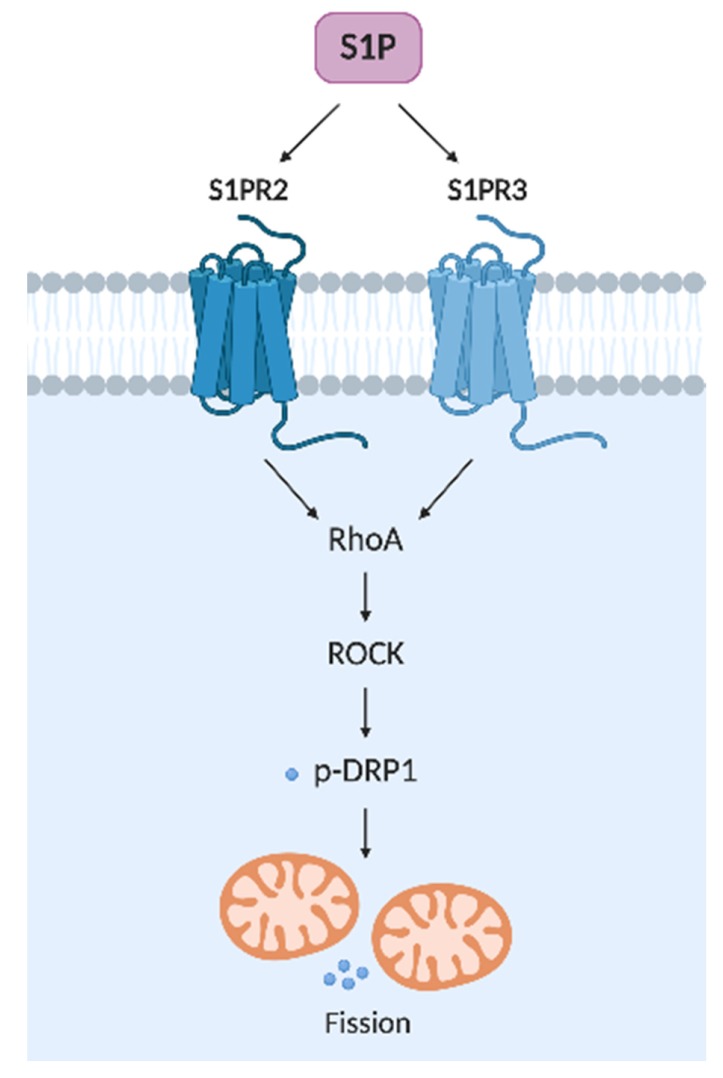
S1P receptors, signaling, and mitochondrial fission. RhoA: Ras homolog family member A; ROCK: Rho-associated protein kinase; p-DRP1: phosphorylated dynamin related protein 1.

As mentioned previously, MFN2 is a protein involved in the mito-fusion process, but it also plays an essential function in the dynamics of the mitochondria-associated membranes (MAMs) [91]. Pulli and co-authors evaluated the sphingosine kinase (SK)1/S1P axis and Ca^2+^ signaling [92]. As MFN2 modulates endoplasmic reticule (ER) and mitochondria interaction, an imbalance in this process is associated with diseases. In the Hela cell line, it was observed that SK1 overexpression significantly augments the IP3-induced release of ER Ca^2+^ that is taken up by the mitochondria. Likewise, the SK1 overexpression induces MFN2 fragmentation, probably by calpain activity. Consequently, SK1 up-regulation increased S1P levels and regulated MFN2 fragmentation associated with an elevation in mitochondrial Ca^2+^ and its downstream cellular effects [92]. The exact role of S1P in mitophagy deserves more studies to understand how S1P/S1PR axis is acting in fission and fusion (Figure 4).

Otherwise, the enzymes regulating S1P balance represent a promising route to understand S1P-regulated autophagic and mitophagy mechanisms [93,94,95]. As known, SGPL1 (sphingosine phosphate lyase 1) catalyzes S1P degradation and has a vital role in S1P-associated cellular functions [96]. The S1P degradation product ethanolamine phosphate (PE) anchors to phagophore membranes for LC3. An SGPL1fl/fl/Nes deficient mouse model has shown a considerable accumulation of S1P and its metabolic precursor sphingosine with no changes in ceramide and sphingomyelin in the brain. When SGPL1 was reduced in the brain, it causes cognitive deficits and a decrease of PE-impaired autophagy, and a consequent accumulation of neurodegenerative biomarkers in SGPL1fl/fl/Nes mice. A reduction in PE leads to the blockage of autophagy flux at the early stages of phagophore formation [97]. It is another work that can support the function of sphingolipids in brain function and potentially related to mitochondria dynamics, highlighting the impacts of these lipids not only on cancer and brain diseases, but other diseases not studied yet.

Furthermore, pathways that regulate mitochondrial function are well conserved from yeast to mammalian cells, as well as sphingolipids metabolism [98,99]. Interestingly, it has been shown that yeast cells lacking inositol sphingolipid phospholipase C (Isc1p), the mammalian ortholog of neutral sphingomielynase type 2 (nSMase), exhibited mitophagy activity enhanced and accumulation of ceramide, which consequently shortened the lifespan and activated Sit4p (PP2A-like protein phosphatase) and Hog1p kinase [100]. Remarkably, these cells had high levels of Dnm1p (Drp1 in mammalian cells), which interact with Isc1p, suggesting that mitochondrial fragmentation is the result of enhanced Dnm1p-mediated fission and showing that Isc1p has a regulatory role in mitochondrial dynamics [100]. This work illustrated that new studies could be performed in different models of organisms to improve the current knowledge of sphingolipids and mitochondria.

## 4. Conclusions and Future Perspectives

Several studies with different types of cells and models support the role of sphingolipids as regulators of mitochondrial dynamics. Overall, the balance of the specific sphingolipids in cells and mitochondria needs to be regulated tightly; once there are several ways to be compromised or modulated such as alterations in gene expression, enzyme activities, receptor functions, polymorphisms, and isoforms of proteins. Therefore, ceramides (C18 and C16) and sphingosine-1-phosphate in mitochondria, as well as CerS1 and ER/mitochondria trafficking, impact the mitochondrial dynamics.

Further studies to unravel the molecular mechanisms and signaling pathways regulating sphingolipids in mitochondria, as well as their specificities in different types of cells and conditions, should contribute to understanding the biological complexity of the sphingolipids and the association between mitochondria, metabolism, and cell homeostasis, with a potential to identify new therapeutic targets in human pathologies such as metabolic disorders and cancer.
